# *Sargassum muticum* and *Osmundea pinnatifida* Enzymatic Extracts: Chemical, Structural, and Cytotoxic Characterization

**DOI:** 10.3390/md17040209

**Published:** 2019-04-03

**Authors:** Dina Rodrigues, Ana R. Costa-Pinto, Sérgio Sousa, Marta W. Vasconcelos, Manuela M. Pintado, Leonel Pereira, Teresa A.P. Rocha-Santos, João P. da Costa, Artur M.S. Silva, Armando C. Duarte, Ana M.P. Gomes, Ana C. Freitas

**Affiliations:** 1 CBQF—Centro de Biotecnologia e Química Fina—Laboratório Associado, Escola Superior de Biotecnologia, Universidade Católica Portuguesa, Rua Diogo Botelho 1327, 4169-005 Porto, Portugal; drodrigues@porto.ucp.pt (D.R.); arpinto@porto.ucp.pt (A.R.C-P.); sdcsousa2@gmail.com (S.S.); mvasconcelos@porto.ucp.pt (M.W.V.); mpintado@porto.ucp.pt (M.M.P.); afreitas@porto.ucp.pt (A.C.F.); 2 Marine and Environmental Sciences Centre (MARE), Department of Life Sciences, Faculty of Sciences and Technology, University of Coimbra, 3000-456 Coimbra, Portugal; leonel.pereira@uc.pt; 3 CESAM–Centre for Environmental and Marine Studies & Department of Chemistry, University of Aveiro, Campus Universitário de Santiago, 3810-193 Aveiro, Portugal; ter.alex@ua.pt (T.A.P.R.-S.); jpintocosta@ua.pt ((J.P.d.C.); aduarte@ua.pt (A.C.D.); 4 QOPNA–Organic Chemistry, Natural Products and Food Stuffs Research Unit & Department of Chemistry, University of Aveiro, Aveiro, 3810-193, Portugal; artur.silva@ua.pt

**Keywords:** *Osmundea pinnatifida*, *Sargassum muticum*, enzymatic extracts, minerals, mono and polysaccharides, FTIR-ATR, NMR, cytotoxicity

## Abstract

Seaweeds, which have been widely used for human consumption, are considered a potential source of biological compounds, where enzyme-assisted extraction can be an efficient method to obtain multifunctional extracts. Chemical characterization of *Sargassum muticum* and *Osmundea pinnatifida* extracts obtained by Alcalase and Viscozyme assisted extraction, respectively, showed an increment of macro/micro elements in comparison to the corresponding dry seaweeds, while the ratio of Na/K decreased in both extracts. Galactose, mannose, xylose, fucose, and glucuronic acid were the main monosaccharides (3.2–27.3 mg/g_lyophilized extract_) present in variable molar ratios, whereas low free amino acids content and diversity (1.4–2.7 g/100 g_protein_) characterized both extracts. FTIR-ATR and 1H NMR spectra confirmed the presence of important polysaccharide structures in the extracts, namely fucoidans from *S. muticum* or agarans as sulfated polysaccharides from *O. pinnatifida*. No cytotoxicity against normal mammalian cells was observed from 0 to 4 mg_lyophilized extract_/mL for both extracts. The comprehensive characterization of the composition and safety of these two extracts fulfils an important step towards their authorized application for nutritional and/or nutraceutical purposes.

## 1. Introduction

Seaweeds are extremely versatile organisms that are widely used for direct human consumption, being considered a food with high commercial value. Furthermore, they are also currently recognized as a huge source of new untapped ingredients, many of which, with biological activity, playing a positive role on health and with great potential to be exploited for food and/or nutraceutical applications. According to Singh and Reddy [1], a wide variety of products derived from seaweeds (including food products) are industrially produced, rendering an estimated total annual value of US $5.5 to 6 billion. Extraction plays a major role in supporting this substantial increase in the importance of seaweeds as a source of new bioactive compounds. Enzyme-assisted extraction (EAE) has gained attention as an effective tool to improve the extraction yield of bioactive compounds from different organisms containing a cell wall, such as seaweeds, while being able to maintain the bioactive properties of the derived extracts [2,3].

Among the edible seaweeds, *Sargassum muticum* (Phaeophyceae) is a brown seaweed of an invasive nature in Europe, containing a high content of antioxidant compounds, such as carotenoids and phenols [4], and therefore its exploitation as a potential food ingredient would be an added value helping to overcome its invasive character. In turn, *Osmundea pinnatifida* (Rhodophyta), an edible red macroalgae, is also found in several parts of European coasts, and recent studies have revealed its huge potential as a food ingredient [5]. Enzymatic extracts from *O. pinnatifida* and from *S. muticum* have demonstrated important biological properties, such as antioxidant, antidiabetic, and prebiotic activities [3]. Therefore, further insights into their chemical and structural properties as well as a potential cytotoxicity evaluation would consolidate the knowledge and safety criteria required for these seaweeds’ enzymatic extracts, with demonstrated biological potential, to be used for food or nutraceutical applications.

To the best of our knowledge, the chemical and structural characterization of *S. muticum* and of *O. pinnatifida* seaweeds’ enzymatic extracts, and the consecutive correlation with previously observed bioactivities [3], has not been performed. Hence, after identifying this important need, the main aim of the research was to determine: i) The elemental, as well as the amino acid and monosaccharide composition for each extract; ii) structural characterization based on FTIR-ATR and ^1^H NMR analysis; and iii) safety validation by assessment of cytotoxicity by testing the metabolic activity of cells when in contact with the extracts. Safety validation as well as structural and chemical elucidation of the underlying multifunctional *O. pinnatifida* and *S. muticum* enzymatic extracts’ roles in nutritional and supplement use were achieved.

## 2. Results and Discussion

### 2.1. Elemental Inorganic and Organic Composition of Seaweeds’ Enzymatic Extracts

The elemental inorganic composition of the *S. muticum* and *O. pinnatifida* enzymatic extracts are shown in Table 1. For the majority of the macro and micro elements evaluated, increments were observed in comparison to the concentrations found in the corresponding dry seaweeds as supported by the respective ratios. Despite the higher contents in minerals, concentration factors thereof, represented by the calculated ratio, differed between both the macro element and extract origin.

The main constituent in both extracts was indeed K and it was also the macro element with the maximum associated ratio in the case of the *S. muticum* extract (ratio of 7.1, Table 1) and the second highest in the case of the *O. pinnatifida* extract (ratio of 6.7, Table 1). In the latter case, the macro element with the largest associated ratio was Mg (ratio of 7.7, Table 1), albeit it had almost a 5-fold lower content (compared to the K content—174 mg/mg_lyophilized extract_). Magnesium levels were in the same range of values for both extracts (29.3–36.8 mg/mg_lyophilized extract)_ and represented the third major macro element found in both extracts (after K and Na); magnesium is known as an important mineral for cardiovascular function. The *S. muticum* extract also enabled a significant concentration of the P macro element with a ratio of 5.1 (Table 1). Phosphorus is essential because it is part of the skeletal structure and teeth, but it also has other important functions, such as its contribution to the control of the acid-base balance in the blood and to carbohydrate metabolism where it, contributes to the intestinal absorption of glucose by the process of phosphorylation [6]. 

Despite the widespread increase in the content of the various macro elements, the ratio of Na/K diminished in both extracts in comparison to the corresponding dry seaweeds. In the case of the *S. muticum* extract, the ratio of Na/K diminished four-fold from 0.65 to 0.16 whereas the three-fold decrease for *O. pinnatifida* extracts was of a larger amplitude, from 3.6 to 1.3. Extracts with a low ratio of Na/K are good candidates to be used as added-value multifunctional salt replacers contributing to the important current trend of salt reduction in food formulation and production.

In both enzymatic extracts, variations in terms of microelements content were observed. In general, *S. muticum* extract was poorer in microelement composition than the *O. pinnatifida* counterpart, namely in Zn, Fe, and Mn contents, which were 10-fold higher in the *O. pinnatifida* extract. Concerning the other microelements, higher variability was observed in both extracts. Noteworthy, there was a high increment of Cu and Mn in *S. muticum* and in *O. pinnatifida* extracts, respectively (Table 1), in comparison to the corresponding dried seaweeds’ contents [5]. The Fe ratio increased 5 times in the *O. pinnatifida* extract (ratio of 4.9, Table 1) in comparison to the dried seaweed, being the major microelement found in this extract. In turn, in the *S. muticum* extract, the Fe ratio decreased slightly relative to the dried seaweed. The importance of Fe for human beings is well-known; Fe is a natural component of several enzymatic systems, being crucial for the transport of oxygen, and its deficiency has been reported as one of the most common nutritional disorders worldwide that may cause anaemia [7].

Regarding the elemental inorganic composition of the enzymatic *S. muticum* and *O. pinnatifida* extracts, some evidences are highlighted: i) Enzymatic extract of *O. pinnatifida* obtained with Viscozyme could be a good contributor of K, Mg, Zn, and Mn (added value) to recommended daily intakes (RDIs) as well as of Fe (similarly to the dried seaweed) whereas *S. muticum* obtained with Alcalase could be a good contributor of K, Mg, and P to RDIs (added value of extract given the concentration factor for P); ii) in general, enzymatic aqueous extraction enables concentrations of the majority of the macro and micro element in both extracts. 

Observing the organic elemental data of both seaweed extracts (Table 1), slightly higher contents of %N and %C were observed for the *S. muticum* enzymatic extract, which was correlated with the higher nitrogen content found in this extract in comparison with the *O. pinnatifida* enzymatic extract [3]. In turn, slightly higher values of %H and %S were observed in *O. pinnatifida* enzymatic extract, which was correlated with the higher contents of polysaccharides, including sulfated polysaccharides, present in this extract in comparison to the *S. muticum* enzymatic extract [3].

### 2.2. Monosaccharides and Free Amino Acids

The composition of monosaccharides, uronic acids, and amino-monosaccharides in seaweed extracts is displayed in Table 2. 

All these compounds play important physiological roles in the original source as well as in the host ingesting these extracts. The most abundant monosaccharides in both seaweed extracts were galactose, mannose, glucuronic acid, and glucosamine. 

Brown marine seaweeds are recognized as a source of complex polysaccharides, such as fucoidans, laminaram, and alginate. For example, fucoidans are made up of glucose, xylose, fucose, mannose, galactose, uronic acids, and acetyl groups, which can also contain some protein components as well as sulfate substituents [8]. 

According to Dore et al., (2013) [9], fucan sulfated polysaccharides extracted from the brown seaweed, *Sargassum vulgare*, were composed of galactose, fucose, xylose, mannose, and glucuronic acid; proportions varied according to the fractions of the fucans extracted. In the *S. muticum* extract obtained with Alcalase, the relative proportions of these monosaccharides found were 1.0:0.22:0.17:0.41:0.91 (Table 2), which provides evidence of the presence of fucoidans in the extract. 

Studies on the *Sargassum* genus concerning fucans shows that they are generally composed of mannose, galactose, and glucuronic acid residues with partially sulfated-chains consisting of fucose, xylose, and galactose [9]. For example, alginate-free aqueous extracts of *S. muticum* had variable relative proportions of glucose, galactose, fucose, xylose, mannose, and uronic acids [8]. In addition, other factors, such as environmental conditions, geographic location, harvest season, species, and life-cycle stage, as well as extraction can have impacts on fucoidans’ composition, structure, and molecular mass [8]. 

Fucoidans from brown seaweeds do not always have the same backbone: In some cases, a backbone of 3-linked α-l-fucopyranose is present, whereas in other cases, the backbone has alternating 3- and 4-linked α-l-fucopyranose residues and sulfated galactofucans [10]. The latter are mainly found in various *Sargassum* species [11]. These are built of (1→2)-β-d-mannose and/or (1→6)-β-d-galactose units with branching points formed by (1→4)-α-d-glucuronic acid, (1→4) and/or (1→3)-α-l-fucose, terminal β-d-xylose, and sometimes (1→4)-α-d-glucose [11].

The presence of glucosamine (Table 2) in the enzymatic extract of *S. muticum* (7.9 mg/g_lyophlized extract_) indicates the presence of a proteoglycan-like material, such as that reported for *Sargassum filipendula* (Phaeophyceae) by García-Ríos et al. [12]. Glucosamine has been studied as an important amino-sugar with beneficial physiological roles in joint health [13]. Although experts have mentioned the need for more research to explore possible beneficial effects of glucosamine in healthy subjects or on risk factors of osteoarthritis, prophylactic evidence has been shown in some animal models. According to a survey of supplements available in the market [14], *S. muticum* extract may be used to develop glucosamine rich powder formulations.

*Osmundea pinnatifida* extract showed higher contents of amino-monosaccharides, uronic acids, and monosaccharides than the *S. muticum* counterpart (Table 2), which parallels the total sugars and sulfated sugars quantified therein. These uphold the potential role of prebiotic activity confirmed by high counts (>10^7^ Log cfu/mL) of viable cells of *Bifidobacterium animalis* subsp. *lactis* BB-12 [3]. The richness of the *O. pinnatifida* extract in galactose and the lower quantities of xylose are in agreement with agaran polysaccharides. Agarans are galactans biosynthesized by red seaweeds constituted by 3-linked β-d-galactose alternating with 4-linked α-l-galactose units presenting different degrees of cyclization of the α-l-galactose residues to give 3,6-anhydro-α-l-galactose [15]. Agarans also present a certain degree of substitution with methyl ethers, pyruvate ketals, sulfate ester groups, d-xylose, and/or 4-*O*-methyl-l-galactose side chains, and different percentages of 3,6-anhydrogalactose [16]. Higher galactose contents with variable proportions of glucose, mannose, xylose, and fucose were reported by Canelón et al. [15] for aqueous extracts of *Laurencia* spp. (red seaweed). No data was found in the literature for red seaweed *O. pinnatifida* extracts. 

Very low contents (1.4–2.7 g/100g_protein_) and a low diversity of free amino acids (aspartic acid, glutamic acid, and methionine) characterized both seaweed extracts. Apparently, the higher nitrogen content in the *S. muticum* extract obtained with Alcalase [3] was not reflected in a higher content of free amino acids, which are probably present in the form of peptides. 

### 2.3. Structural Characterization of Seaweed Extracts

**Figure 1:**
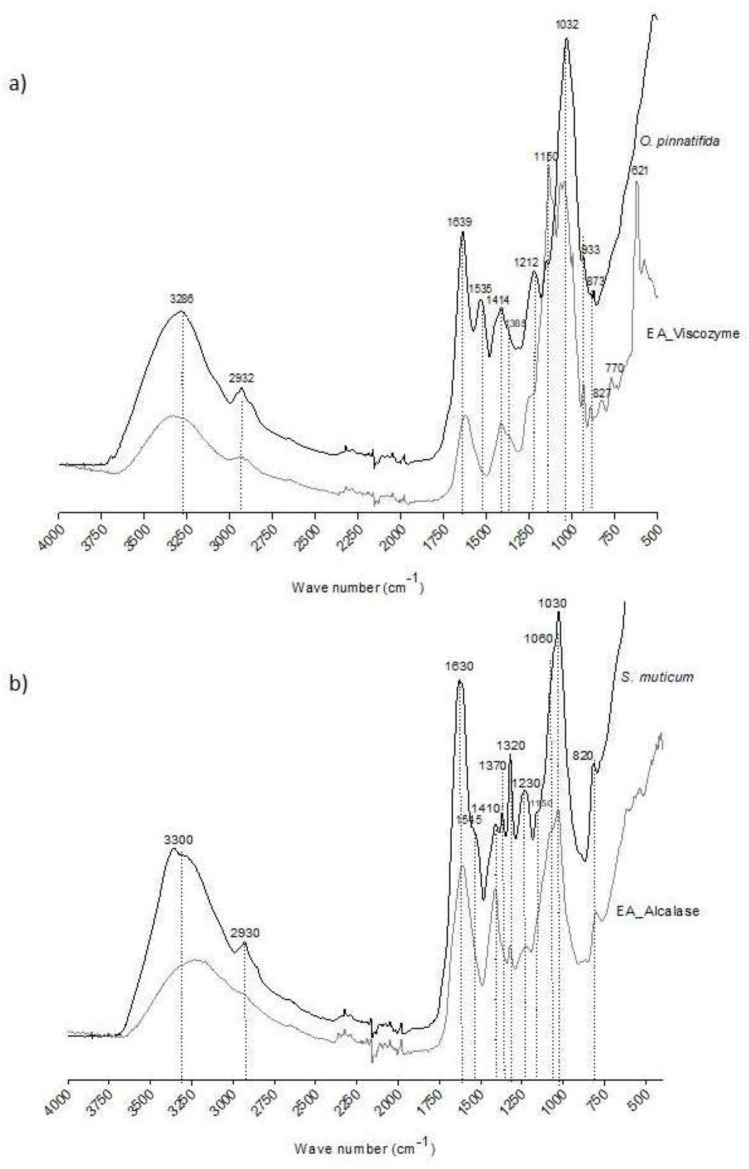
FTIR-ATR spectra of the red edible seaweed, *O. pinnatifida*, and of its enzymatic extract obtained by Viscozyme (**a**) and of the brown edible seaweed, *S. muticum*, and of its enzymatic extract obtained by Alcalase (**b**).

The FTIR-ATR spectra of *S. muticum* and its enzymatic extract are displayed in Figure 1a. Both spectra reveal high similarity with most of the bands being common between *S. muticum* seaweed and corresponding extract. Practically, differences were only observed in the absorption intensity. N-H stretching vibrations at 3700–2900 cm^−1^ as well as from amide I and amide II at 1700–1420 cm^−1^ are present in both spectra and could be related to proteins; this is in agreement with the previous results for the nitrogen content [3].

A broad band at 3280–3350 cm^−1^ and a weaker signal at 2870–2960 cm^−1^ could be assigned to O-H and C-H stretching vibrations, but also to N-H stretching vibrations, respectively [17]. The two characteristic absorptions, a band at around 1630 cm^−1^ (C-O asymmetric stretching vibration) and a band of 1410 cm^−1^ (C-O symmetric stretching vibration) [18], indicates the presence of carboxyl groups in both *S. muticum* seaweed and in its extract. In the *S. muticum* Alcalase extract spectrum, there is a noteworthy intensity increment of the 1410 cm^−1^ band in comparison to other bands. This could indicate the higher presence of protein or peptides in the extract due to the role of the endopeptidase, Alcalase. This enzyme was, in fact, responsible for a significantly higher nitrogen content (*p* < 0.05) in the *S. muticum* extract [3], but lower contents of free amino acids were detected in this extract.

A focus on the 700–1400 cm^−1^ region is important because it is related to the seaweeds’ polysaccharides, namely carrageenan and agar in red seaweeds and alginates and fucoidans in brown seaweeds. Alginate is a polysaccharide, which has been found in brown seaweeds, such as *S. muticum*, known to be a linear copolymer of β-D-mannuronic acid and α-l-guluronic acid (1-4)-linked residues arranged in heteropolymeric and/or homopolymeric blocks. The presence of these acids can be evidenced especially by the bands around 1030 and 1060 cm^−1^ assigned to guluronic acid and at 1320 cm^−1^ assigned to mannuronic acid, all present both in *S. muticum* seaweed and in its extract obtained by Alcalase. In terms of the broad band around 1220–1260 cm^−1^ in the FTIR-ATR spectrum, assigned to the presence of sulfate ester groups (S=O), which is a characteristic component in fucoidan and other sulfated polysaccharides that can be found in some brown seaweeds [17,19], it is particularly observable in the *S. muticum* seaweed spectrum and less in its extract obtained by Alcalase (Figure 1a). These results are in agreement with the monosaccharide compositions observed previously for *S. muticum* extract, which evidences the presence of fucoidans (Table 2). Fucoidans have been reported as being responsible for some α-glucosidase inhibitory activity from fractions of *Sargassum duplicatum* [20]. The α-glucosidase inhibitory activity was also observed for *S. muticum* extracts obtained with Alcalase [3]. 

In terms of the *O. pinnatifida* seaweed and its enzymatic extract obtained by Viscozyme, a multi-enzyme complex of carbohydrases (arabanase, cellulase, β-Glucanase, hemicellulase, and xylanase), the two spectra presented some qualitative differences in the region of 1100 to 1600 cm^−1^ (Figure 1b). In the extract spectrum, the bands at 1535 and 1212 cm^−1^ almost disappeared whereas the band at 1150 cm^−1^ increased in comparison to the *O. pinnatifida* seaweed spectrum. 

The absorbance bands at 1222 and 1150 cm^−1^ are characteristic of less sulfated polysaccharides, such as agar. Strong absorption at 1220 to 1260 cm^−1^ have been reported by Yu et al., (2012) [21] for agaran-type polysaccharides isolated from *Grateloupia filicina* (Rhodophyta), and according to Rodrigues et al. [5], O. *pinnatifida* was considered a red seaweed agar-like producer. The increment in the band at 1150 cm^−1^ may be related to the possible role of the multi-enzyme complex of carbohydrases on matrix polysaccharides (agar) and on cellulose, xylan, and manan fibrils of the complex composite cell walls of red seaweeds. The extract of *O. pinnatifida* was characterized by the highest content of sulfated sugars [5]. The absorbance band at 930 cm^−1^ in both spectra was assigned to the presence of 3,6-anhydro-d-galactose found in carrageenan and agar polysaccharides [22]. These results are in agreement with the monosaccharide compositions observed previously for *O. pinnatifida* extract, which evidences the presence of agaran polysaccharides (Table 2). 

Since the region of 1700 to 1420 cm^−1^ was attributed to amide I and amide II, which in turn could be related to protein, the high reduction of the absorbance band at 1535 cm^−1^ is probably due to some loss of nitrogen content during the extraction process. This correlates well with the fact that enzymatic extracts of *O. pinnatifida* were characterized by low nitrogen contents [5] and low contents of free amino acids.

**Figure 2:**
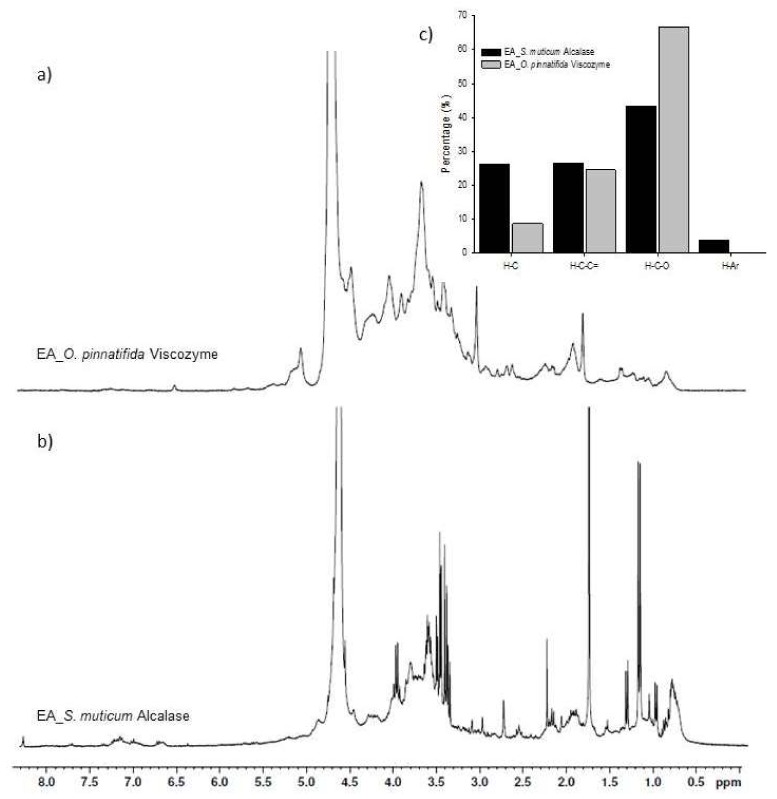
^1^H NMR spectra of enzymatic extracts of *Osmundea pinnatifida* obtained by Viscozyme (**a**) and *Sargassum muticum* obtained by with Alcalase (**b**) (the peak at 4.7 ppm indicates the water signal), as well as the relative abundance of each type of proton (**c**) estimated as the partial integrals of the spectra reported in Figure 2a,b for the enzymatic extracts of seaweeds where H-C: purely alkylic hydrogen atoms; H-C-C=: allylic (H-C_α_-C=), carbonyl or imino (H-C_α_-C=O or H-C_α_-C=N) groups; H-C-O: aliphatic C-H directly bound to an oxygen atom; H-Ar: aromatic hydrogen atoms.

The ^1^H NMR spectra of both seaweeds’ enzymatic extracts were quite similar (Figure 2a,b).

These spectra reveal distinct peaks overlaying much broader bands, an expected observation given the nature of the NMR spectra of complex mixtures of organic compounds [23]. Despite the large variety of overlapping resonances, each ^1^H NMR spectrum was investigated on the basis of the chemical shift assignments described in the literature for organic compounds [23,24]. Accordingly, four main regions of chemical shifts were considered in each spectrum:

1) *δ*_H_ = 0.6–1.8 ppm: Aliphatic protons, H–C; -CH > -CH_2_ > -CH_3_.
2) *δ*_H_ = 1.8–3.2 ppm: Protons bound to carbon atoms in the alpha position to unsaturated groups in allylic (H-C_α_-C=), carbonyl, or imino (H-C_α_-C=O or H-C_α_-C=N) groups, and protons in secondary and tertiary amines (H-C-NR_2_ and NR_3_, respectively).
3) *δ*_H_ = 3.2–4.1 ppm: Aliphatic protons on carbon atoms singly bound to oxygen atoms (H-C-O-CO-R > H-C-OH or H-C-O-C) in alcohols, polyols, ethers, and esters.
4) *δ*_H_ = 6.5–8.5 ppm (aromatic protons).

In order to further understand the ^1^H NMR data, each spectral region was quantitatively integrated so that the abundance of each of the different types of protons in the different extracts could be assessed; the results are depicted in Figure 2c. Anomeric protons of glycosidic structures [25] related with the fifth region (*δ*_H_ = 4.1–6.0 ppm) were also considered, but not integrated since the wide and intense peak at 4.7 ppm is due to the water signal. 

In accordance with the spectra, the relative abundance of each type of protons is, in general, relatively similar to the different extracts, but some points are worthy of being highlighted. The higher percentages of protons belong to the group of aliphatic H-C directly bound to an oxygen atom (H-C-O) probably due to the presence of nonaromatic ring structures, such as sugars [24]. Higher values (67%) are observed for the enzymatic extract of *O. pinnatifida* obtained with Viscozyme than for the enzymatic extract of *S. muticum* (43%) obtained with Alcalase. These values are in agreement with previously discussed trends for the FTIR-ATR spectra and sugars: Total sugar and sulfated sugar contents found in the *O. pinnatifida* enzymatic extract were 2.3 and 8-fold higher than in the *S. muticum* enzymatic extracts, respectively [3]. According to Bubb [26], ^1^H spectra of carbohydrates do contain some well-resolved signals, including those of anomeric protons (*δ*_H_ = 4.4–5.5 ppm), acetyl (~*δ*_H_ = 2.0–2.1 ppm) and methyl (~*δ*_H_ = 1.2 ppm) groups as well as other protons that are influenced by specific functionality, including amino groups, phosphorylation, sulfatation, glycosylation, and acetylation, or lack of functionality as in deoxy-sugars. Signals due to polyols were described by Tanniou et al. [27], who studied the biochemical composition of *S. muticum* populations by ^1^H HRMAS (high resolution magic angle spinning) NMR and assigned chemical shifts to polyols (mannitol) at *δ*_H_ = 3.5–4.0 ppm.

Not many differences were observed for protons in the alpha position to unsaturated groups in allylic (H-C_∞_-C=), carbonyl, or imino (H-C_∞_-C=O or H-C_∞_-C=N) groups for both seaweed extracts, but the slightly higher value for the enzymatic extract of *S. muticum* (27%) could be related to Alcalase activity, which lead to a 2.4 times higher content of nitrogen compounds in comparison to the enzymatic extract of *O. pinnatifida* obtained with Viscozyme [3]. According to Gonzaga et al. [25], a chemical shift at 2.78 ppm could be assigned to protein groups, which was visible in the spectrum of the *S. muticum* enzymatic extract, but not in the corresponding *O. pinnatifida* enzymatic extract (Figure 2a,b). Proteins were described as being associated to cell wall polysaccharides, being part of the structure of the seaweed cell walls [28]. 

In terms of purely alkylic hydrogen atoms (H-C; 26%) and of aromatic hydrogen atoms (H-Ar; 4%), the enzymatic extract of *S. muticum* was richer (26 and 4%, respectively) in these atoms than the enzymatic extract of *O. pinnatifida* (8.9 and 0.2%, respectively) (Figure 2c). Signals at *δ*_H_ = 7.2–7.3 ppm and *δ*_H_ = 7.6–7.8 ppm (Figure 2a,b) are consistent with the presence of aromatic units containing both electron-donor (e.g., phenolic groups) and electron-acceptor (e.g., carbonyl and carboxyl) substituents. The content of phenolic contents in aqueous extracts was shown to be low; albeit contents of 290 and 123 µg_cathecol equiv_/_glyoph extract_ were determined in the enzymatic extracts of *S. muticum* and *O. pinnatifida* with some potential antioxidant activity, respectively [3]. Signals due to the presence of unsaturated fatty acids were described by Tanniou et al. [27] in *S. muticum* at *δ*_H_ = 1.0–1.5 ppm.

In the anomeric spectral region (*δ*_H_ = 4.1–6.0 ppm), different patterns were observed for both seaweed extracts (Figure 2a,b). In the case of the enzymatic extract of *O. pinnatifida*, signals in the region of *δ*_H_ = 4.2–4.5 and ~*δ*_H_ = 5.1 ppm could be assigned to α and β reducing end units whereas no particular signals were well resolved in this region for the enzymatic extract of *S. muticum*. According to Barros et al. [29], the signal from an anomeric proton at *δ*_H_ = 5.13 was assigned to 3,6-α-l-anhydrogalactose while the signal at *δ*_H_ = 4.56 was attributed to β-d-galactose for polysaccharides of a red seaweed, *Crassiphycus caudatus* (formerly *Gracilaria caudata*). No literature references were found for the ^1^H NMR spectra *O. pinnatifida* extracts. According to Llanes et al. [30], the absence of signals in the regions of *δ*_H_ = 5.0–5.3 ppm and *δ*_H_ = 4.7–4.9 ppm expected for anomeric protons of α and β reducing end units that are released (d-anomeric protons of mannuronosyl and l-guluronosyl residues from hydrolysis of sodium alginate from *Sargassum* sp.) are an indication of limited hydrolysis of the *Sargassum* alginate or resulted from overlapping resonance with the water signal at 4.7 ppm. 

### 2.4. Cytotoxicity Evaluation

**Figure 3:**
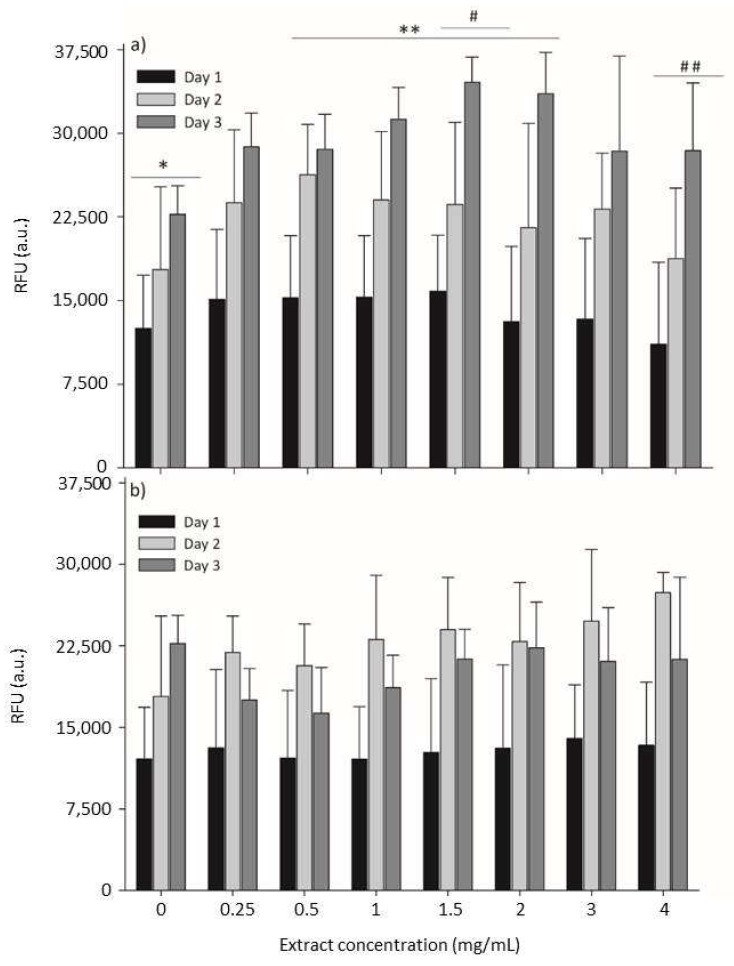
Metabolic activity by the resazurin assay on mouse lung fibroblast cells (L929) cells exposed to different concentrations of enzymatic extracts of *Osmundea pinnatifida* (**a**) and *Sargassum muticum* (**b**). Time of exposure was statistically significant (*p* < 0.05) for each concentration of extract of both seaweeds. Concentration of extract was not statistically significant (*p* > 0.05) for *S. muticum* extracts; for *O. pinnatifida* extracts, statistically significant differences were obtained between 0 mg/mL (*) and 0.5–2.0 mg/mL (* *) and between 1.5 mg/mL (#) and 4.0 mg/mL (# #), respectively.

In order to consolidate the safety of both seaweed extracts, the cytotoxicity of *O. pinnatifida* and *S. muticum* extracts was assessed by measuring the cellular metabolic activity of mammalian mouse fibroblasts cultured with increasing concentrations of both extracts. A range of concentrations from 0 to 4 mg_lyophilized extract_/mL was tested. The results are summarized in Figure 3, where cell metabolic activity was evaluated by the resazurin assay. Resazurin (7-Hydroxy-3*H*-phenoxazin-3-one 10-oxide) is a blue and non-fluorescent compound, which is reduced by viable cells in the presence of mitochondrial NADPH dehydrogenases into pink and highly fluorescent resorufin [31]. After applying the resazurin assay, L929 cells were able to produce high amounts of the resorufin compound upon incubation with different concentrations of both seaweed extracts, which reveals normal cell metabolism and mitochondrial integrity/activity that can be inferred as a direct measure of cell viability.

Upon exposure to increasing concentrations of seaweed extracts, cells did not present cytotoxic behavior (Figure 3), indicating that both extracts do not provoke a detrimental effect on the metabolic activity of cells. 

In contrast, the supplementation of the culture medium with the extracts seemed to promote an increase of cell viability, which was especially noticeable for the *O. pinnatifida* extracts (Figure 3a). When compared to the viability of cells cultured in the absence of extracts (0 mg_lyophilized extract_/mL), cells cultured in the presence of the different concentrations of these extracts demonstrated equal or higher metabolic activity in comparison to the negative control (culture medium only), which can be attributed to the supplementation of the culture media with nutrients from the extracts. For the *O. pinnatifida* extracts, the time of exposure increased the cell metabolic activity for all tested concentrations (Figure 3a), while for *S. muticum*, a slight decrease was observed after 72 h of exposure (Figure 3b). 

Consumer oriented applications, such as biomedical, pharmaceutical, or food applications, are highly demanding in terms of the cytotoxic evaluation for new compounds [32]. Cytotoxic evaluation of seaweed-based compounds [33,34,35] is under-exploited in the literature, and given their significance, this study is of key importance. The anti-tumorigenic effects of compounds extracted from seaweeds were reported [36,37], but it is also highly important to study the effects of these ingredients on normal mammalian cells, an added-value of this study.

## 3. Materials and Methods

### 3.1. Seaweed Extracts

Specimens of the red seaweed (Rhodophyta, Florideophyceae), *Osmundea pinnatifida* (Ceramiales) Rhodomelaceae family, and of the brown seaweed (Heterokontophyta, Phaeophyceae), *Sargassum muticum* (Fucales) Sargassaceae family, were harvested in Buarcos bay (Figueira da Foz, Portugal), and cleaned and dried according to Rodrigues et al., (2015b) [5]. Enzymatic extraction of *O. pinnatifida* by Viscozyme L (Sigma-Aldrich, St. Louis, MO, USA) and of *S. muticum* by Alcalase (Sigma-Aldrich) was performed according to procedures described by Rodrigues et al., (2015a) [3]. In this study, the authors used lyophilized aliquots of the same extracts obtained by Rodrigues et al., (2015a) [3] in order to enable accurate comparison and correlation analyses. 

### 3.2. Chemical Characterization

#### 3.2.1. Elemental Analysis

Determination of the inorganic elements, Mo, B, Zn, P, Cd, Co, Ni, Mn, Fe, Mg, Ca, Cu, Na, Al, and K, in lyophilized extracts was performed in two steps: Microwave-assisted digestion followed by determination of the 15 elements using an inductively coupled plasma (ICP) optical emission spectrometer (OES) with radial plasma configuration according to Rodrigues et al., (2015b) [5]. Three replicates were performed for each sample as well as blanks. 

The organic elements, C, H, S, and N, in lyophilized extracts were quantified using a Truspec 630-200-200 Elemental Analyser (Mönchengladbach, Germany). Triplicate samples up to 3 mg for each extract were placed under combustion at 1075 °C. Carbon, H, and S were detected by infrared absorption whereas N was detected by thermal conductivity.

#### 3.2.2. Analysis of Monosaccharide Composition

Monosaccharide composition was analyzed by high performance liquid chromatography coupled to a UV detector (HPLC-UV, Agilent 1100, Waldbronn, Germany,) after acid hydrolysis. For each lyophilized extract, 2.5 mg of sample was hydrolyzed with 2 mL of 2 M trifluoroacetic acid at 110 °C for 4 h. The hydrolysate was then dried by vacuum evaporation at 50 °C and re-dissolved in 2 mL of deionized water. The hydrolysate solution (450 µL) was mixed with 450 µL of 1-phenyl-3-methyl-5-pyrazolone solution (0.5 M in methanol) and 450 µL of NaOH solution (0.3 M) and then reacted at 70 °C for 30 min. The reaction was stopped by neutralizing with 450 µL of 0.3 M HCl, and the product was then partitioned with chloroform three times. The aqueous layer was collected and filtered through a 0.45 µM membrane and was applied to HPLC. 

The HPLC analysis was performed using a ZORBAX ECLIPSE XDB-C18 column (Agilent) (4.6 × 150 mm, 5 micro) at 25 °C with potassium phosphate buffer saline (0.05 M, pH 6.9) with 15% (solvent A) and 40% acetonitrile (solvent B) as mobile phases and detected by a UV detector at 250 nm. All analyses were made in quintuplicate and quantified using a calibration curve built with monosaccharide standards (Sigma Aldrich, St. Louis, MO, USA) and expressed as mg/g_lyophilized extract_. Glucose, galactose, arabinose, fucose, mannose, xylose, rhamnose, glucuronic acid, galacturonic acid, and glucosamine-6-phosphate were used as the standards. Recovery ranged between 93% and 99% with an LOD of 0.095 mg/g. 

#### 3.2.3. Analysis of Amino Acids

The free amino acid contents of each extract was performed by pre-column derivatization with the orthophthalaldehyde (OPA) methodology. Isoindole-type fluorescent derivatives were formed in an alkaline solution (borate buffer pH 10.4) from OPA, 2-sulfanylethanol, and the primary amine group of the amino acid. The derivatives were separated by HPLC (Beckman Coulter, California USA) coupled to a fluorescence detector (Waters, Milford. MA, USA) according to the procedure of Proestos et al., (2008) [38]. Of each sample, 100 µL, at concentration of 10 mg mL^−1^, was derivatized according to the OPA method and the injection volume of derivatives was 20 µL. All analyses were made in triplicate and quantified using a calibration curve built with amino acid pure standards (Sigma Aldrich, St. Louis MO, USA) and expressed as g/100 g of protein content. Recovery ranged between 92% and 99% with an LOD of 0.02 g/100 g of protein content. 

#### 3.2.4. FTIR-ATR Analysis

Samples of lyophilized extracts were analyzed by Fourier Transform Infrared Spectroscopy with attenuated total reflectance (FTIR-ATR) (Spectrum 100, PerkinElmer, Shelton, CT, USA) according to procedures described in Rodrigues et al., (2015b) [5].

#### 3.2.5. ^1^H NMR Analysis

Twenty milligrams of each lyophilized extract were suspended in 700 μL of D_2_O and homogenized for 10 min in a vortex. Then, an amount of dissolved lyophilized extract (650 μL) was placed in 5 mm NMR tubes (Sigma-Aldrich, 528-PP, 5 mm). 

All spectra were acquired on a Bruker Advance 300 spectrometer (Karlsruhe, Germany) with an operating frequency of 300.13 MHz. Spectra were acquired with a spinning rate of 20 Hz, a contact time of 4.75 s, and with the pulse program, ZG30. The recycle delay was 1 s and the length of the proton 90 pulses was 9.00 μs. About 56 scans were collected for each spectrum. A 0.3 Hz line broadening weighting function and a baseline correction were applied. The identification of functional groups in the NMR spectra was based on their chemical shift (δ_H_) relative to that of the water (4.7 ppm).

### 3.3. Cytotoxicity Assessment

Mouse lung fibroblast cell line (L929) was used to assess cytotoxicity. Cells were maintained in Dulbecco’s Modified Eagle’s Medium (DMEM) (Lonza, Verviers, Belgium) supplemented with 10% fetal bovine serum (FBS) (Lonza, Verviers, Belgium) and 1% of antibiotic–antimycotic mixture (Biowest, Nuaillé, France). The cells were grown at 37 °C and 5% CO_2_ in a humidified incubator. Exponentially growing cells were used throughout the experiment. TrypLE™ Express Enzyme (Gibco, New York, NY, USA) was used to detach the cells. 

For each cell culture assay, the cells were seeded in 48-well plates (*n* = 3) at a concentration of 2 × 10^4^ cells/well and allowed to attach for 24 h at 37 °C and 5% CO2. After that time, cells were washed with phosphate buffered saline (PBS) and exposed for 24, 48, and 72 h to enzymatic extracts of *O. pinnatifida* and *S. muticum* containing different concentrations (0, 0.25, 0.5, 1, 1.5, 2, 3, and 4 mg/mL). All extracts were previously prepared using DMEM and filtered using Vacuum Filtration rapid-Filtermax (PES 0.22 µL membranes) (Trasadingen, Switzerland). Dimethyl sulfoxide (DMSO 20%) was used as a positive control of cell death, due to its strong cytotoxic effect. Culture medium was used as a negative control of cytotoxicity, considered to be the ideal situation for cell growth. At each time point, exposed and control wells were washed with PBS and cell metabolic activity was assessed using the resazurin assay [31]. Cells with 20% *v*/*v* of stock resazurin solution (1 mg/mL, Sigma) in culture medium were incubated for 2 h at 37 °C. Afterwards, 100 µL of supernatants were transferred to a 96-well black plate (Greiner) and the relative fluorescence units (RFU) were measured using a microplate reader, SynergyTM Mx HM550 (BioTek^®^ Instruments, Vermont, NH, USA), set at 530/590 nm (excitation/emission wavelength, respectively). The obtained results were normalized by subtraction of the negative control (without cells). Samples were measured in triplicate and experiments repeated at least three times.

### 3.4. Statistical Analysis

Two-way analysis of variance (ANOVA) was carried out for cytotoxicity assessment of both extracts, with SigmaStat™ (Systat Software, Chicago, IL, USA) to assess if the concentration of the extract and time of exposure were significant sources of variation for metabolic activity (resazurin assay) of L929 fibroblasts. For each ANOVA, it was verified that both the normality and equal variance tests were non-significant (*p* > 0.05) as well as interactions between the concentration and time (*p* > 0.05). The Holm-Sidak method was used for pair-wise comparisons both for concentration and time effects.

## 4. Conclusions

The extensive chemical, structural, and cytotoxicity characterization of *S. muticum* and *O. pinnatifida* enzymatic extracts enabled a deeper understanding and justification for their previously identified multifunctionality. Analysis of the elemental inorganic composition of the seaweeds showed that enzymatic aqueous extraction enabled an important concentration effect of almost all macro and micro elements in comparison to the dry seaweeds’ contents, and in some cases, the nutritional value (an extract containing at least 15% of the mineral RDI value) was enhanced, in particular for K and P in the *S. muticum* enzymatic extract and for K, Mg, Zn, and Mn in the *O. pinnatifida* extract.

Overall, the higher contents of monosaccharides, uronic acids, and glucosamine, which were observed in the *O. pinnatifida* extract obtained with Viscozyme in comparison to the *S. muticum* extract, were in agreement with the total sugars and sulfated sugars previously quantified in these extracts, and were further well correlated with the structural analysis obtained from the FTIR-ATR and ^1^H NMR spectra. The results highlight the relevance of such characterization since both seaweed extracts showed a diversity of sugars in variable molar ratios. On the other hand, amino acids were not representative both qualitatively and quantitatively.

According to chemical and structural analysis by FTIR-ATR and ^1^H NMR, both extracts, with prebiotic and antidiabetic potential, are composed of important polysaccharide structures, confirming, for example, fucoidans in *S. muticum* extract or agarans as sulfated polysaccharides in *O. pinnatifida* extract among the main representative polysaccharides. No cytotoxicity against normal mammalian cells was observed, making these seaweed extracts very interesting functional ingredients, which could be explored as a food ingredient (salt replacer, nutrient vector) or nutraceutical supplement.

## Figures and Tables

**Table 1:** Elemental inorganic and organic composition of enzymatic seaweeds’ extracts.

			EA_*S. muticum* Alcalase		EA_*O. pinnatifida* Viscozyme	
			(mg/mg_lyophilized extract_)	Ratio ^1^	(mg/mg_lyophilized extract_)	Ratio ^1^
Inorganic	Macro elements	K	407 ± 13	7.1	174 ± 3	6.7
		Na	66 ± 2	1.8	222 ± 3	2.4
		Ca	2.31 ± 0.06	0.3	8.34 ± 0.04	1.5
		Mg	29.3 ± 0.3	2.0	36.8 ± 0.8	7.7
		P	11.6 ± 0.5	5.1	6.30 ± 0.09	3.6
	Micro elements	Zn	0.033 ± 0.002	1.3	0.34 ± 0.02	5.9
		B	0.319 ± 0.004	3.0	0.28 ± 0.01	2.2
		Mn	0.045 ± 0.004	4.1	0.34 ± 0.06	29.2
		Fe	0.17 ± 0.02	0.9	1.8 ± 0.1	4.9
		Al	<LOD	-	0.085 ± 0.07	0.6
		Cu	0.065 ± 0.002	14.4	0.026 ± 0.001	5.3
		Ni	<LOD	-	0.20 ± 0.01	-
		Pb	0.040 ± 0.001	-	0.020 ± 0.001	-
Organic		%N	2.5	-	1.3	-
		%C	17.1	-	16.8	-
		%H	2.5	-	3.5	-
		%S	0.7	-	1.7	-

^1^ Ratio = content in lyophilized extract/content in dry seaweed; values of organic elements are presented as average of triplicate samples. LOD: Limit of Detection.

**Table 2:** Composition of monosaccharides, uronic acids, and amino-monosaccharide in enzymatic extracts of the seaweeds, *S. muticum* and *O. pinnatifida*.

		*S. muticum*_Alcalase (mg/g_lyophyzed extract_)	*O. pinnatifida*_Viscozyme (mg/g_lyophyzed extract_)
Monosaccharides	Glucose	<LOD	<LOD
	Galactose	19.1 ± 0.3	25.3 ± 0.2
	Mannose	7.8 ± 0.2	11.4 ± 0.2
	Arabinose	0.10 ± 0.01	0.16 ± 0.01
	Xylose	3.23 ± 0.02	4.8 ± 0.1
	Rhamnose	0.27 ± 0.01	0.52 ± 0.01
	Fucose	4.3 ± 0.1	5.60 ± 0.09
Uronic acids	Glucuronic acid	17.4 ± 0.3	27.3 ± 0.2
	Galacturonic acid	1.07 ± 0.01	1.50 ± 0.02
Amino-mon.	Glucosamine	7.9 ± 0.1	12.7 ± 0.4
